# Synergistic effects of dioscin and amphotericin B against *Candida albicans*: a promising strategy for antifungal therapy

**DOI:** 10.3389/fcimb.2025.1730185

**Published:** 2026-01-05

**Authors:** Xin Liu, Fei Xu, Yu Zhang, Lili Zhong, Longfei Yang

**Affiliations:** 1 Eye Center, The Second Hospital of Jilin University, Changchun, China; 2 Department of Acupuncture and Moxibustion, The Second Hospital of Jilin University, Changchun, China; 3 Department of Clinical Laboratory, The Second Hospital of Jilin University, Changchun, China; 4 Jilin Provincial Key Laboratory on Molecular and Chemical Genetics, The Second Hospital of Jilin University, Changchun, China

**Keywords:** *Candida albicans*, dioscin, amphotericin B (AmB), antifungal, synergy, virulence factors

## Abstract

The emerging antifungal resistance exacts deteriorating effects on the availability of drugs for treating fungal infections, calling for novel therapies. Although amphotericin B (AmB) is clinically effective and rarely causes resistance, its severe side effects warrant further reduction. Enhancing or potentiating AmB efficacy with other agents to lower the dose of AmB is appealing to reduce AmB toxicity. Dioscin, a steroidal saponin from the *Dioscorea* genus, has shown multiple pharmacological activities, including anticancer, antifungal, hepatoprotective, and nephroprotective effects. As combinational therapy has multiple advantages, it is interesting to explore the effects of dioscin and AmB combination in *C. albicans*, which is the most common fungal pathogen in humans. In this study, through a checkerboard assay, we found that dioscin and AmB produced synergistic effects in inhibiting the planktonic growth of *C. albicans*, *Candida krusei*, and *Candida tropicalis* at 24 h. Dioscin (1 μg/mL) and AmB (0.625 μg/mL) synergistically inhibited the biofilm formation (97%) and development (60%) of *C. albicans* (SC5314), significantly superior to either agent alone (*p* < 0.01). Time-killing assay revealed that the combination of dioscin (1 μg/mL) and AmB (0.625 μg/mL) greatly enhanced the killing efficacy compared to either agent alone. The hyphal formation and adhesion to abiotic surfaces of *C. albicans* were also suppressed by this combination. The damages to the cell membrane caused by this combination were revealed by the cell membrane integrity assay and cell membrane potential assay using fluorescent dyes. Cell membrane damage was further confirmed by results from the transmission electron microscope and scanning electron microscope. Overall, our results suggested that the synergistic combination of dioscin and AmB causes cell membrane damage and holds great potential for future development as anti-*Candida* therapies.

## 1 Introduction

Among the fungi that can cause infections in humans, *Candida albicans* is the most common opportunistic fungal pathogen and can infect mucosae and skin of immunocompromised patients (Liu et al., 2017; Muthamil et al., 2018). Once this fungus enters the bloodstream, probably through gastrointestinal tract breaches or implantable medical devices (such as catheters), life-threatening candidemia can occur, the mortality of which is as high as 40% despite antifungal therapies (Han et al., 2019; Liu et al., 2017; Russell et al., 2023). *C. albicans* can also form refractory biofilms on biotic or abiotic surfaces, such as oral mucosa and surfaces of biomedical materials, respectively (Liu et al., 2017). Current antifungal drug development has lagged behind the incidence of antifungal drug resistance and the emergence of new fungal pathogens. In this scenario, the reasonable application of currently available antifungal drugs is vital for public health management. This includes curbing the development of resistance to current antifungal drugs by lowering the antibiotics abuse and employing a combinational strategy to enhance or potentiate the efficacy of antifungal drugs. Combinations using phytochemicals and conventional antifungal drugs have several advantages, such as enhanced efficacy, decreased dose (thus lowering the drug toxicity), and lowered emergence of drug resistance (Muthamil et al., 2018; Revie et al., 2022). Examples include ginkgolide B and fluconazole (Li et al., 2020), artemisinin and amphotericin B (AmB) (Zhu et al., 2021), and pseudolaric acid A and fluconazole (Zhu et al., 2022).

Dioscin is a saponin that can be isolated from many medicinal and edible plants, including *Dioscorea nipponica*, *Dioscorea cayenensis*, and *Dioscorea alata* (Cho et al., 2013; Sautour et al., 2004; Tang et al., 2013; Yang et al., 2019b). This saponin has been shown to have antifungal activities against human fungal pathogens, including *C. albicans*, *Candida tropicalis*, *Candida glabrata*, *Candida parapsilosis*, *Trichosporon beigelii*, and *Malassezia furfur* (Cho et al., 2013; Sautour et al., 2004; Yang et al., 2018a). The antifungal activity of dioscin involves damage to the cell membrane, which is also related to its inhibitory effects on *C. albicans* biofilms (Cho et al., 2013; Yang et al., 2018a). As the typical antifungal drug AmB also targets the cell membrane to exert its fungicidal activity, it is interesting to test the efficacy of the combination of dioscin and AmB against *C. albicans*, which poses a threat to public health (Liu et al., 2017). The purpose of this study was to explore whether dioscin can synergize with AmB in inhibiting the growth and virulence factors of *C. albicans*, as well as to investigate the underlying mechanisms.

## 2 Materials and methods

### 2.1 Strains and chemicals

*C. albicans* SC5314 and other *Candida* standard strains were obtained from the China General Microbiological Culture Collection Center (CGMCC, Beijing) (Yang et al., 2024b). The *C. albicans* clinical isolates were provided by the Clinical Laboratory of the Second Hospital of Jilin University (Yang et al., 2024a). These strains were kept on yeast extract–peptone–dextrose (YPD) agar at 4 °C in a refrigerator, and before each assay, one colony was transferred into YPD liquid medium for overnight culture at 28 °C with a rotation of 140 rpm. AmB and 2,3-*bis*(2-methoxy-4-nitro-5-sulfophenyl)-2H-tetrazolium-5-carboxanilide (XTT) were purchased from Sangon Biotech (Shanghai, China). Dioscin (SD8350) was purchased from Solarbio (Beijing, China). Propidium iodide (PI) was from Sigma Aldrich (Shanghai, China). *Bis*(1,3-dibutylbarbituric acid) trimethine oxonol (DiBAC_4_(3)) was purchased from MCE Company (Shanghai, China).

### 2.2 Antifungal susceptibility assay

Checkerboard method and CLSI-M27-A3 guidelines were followed to determine the combinational effects of dioscin and AmB, as described previously (Yang et al., 2019a). Briefly, *Candida* cells from overnight cultures were collected through centrifugation (3,000×*g*, 5 min) and diluted to 2 × 10^3^ cells/mL in Roswell Park Memorial Institute (RPMI) 1640 medium. A total of 100 μL of such cell suspension was added to each well of a 96-well plate, followed by the addition of dioscin (0.125–8 μg/mL) and AmB (0.156–10 μg/mL) using a two-times serial dilution. After 24-h incubation at 37 °C, the minimal inhibitory inhibition (MIC) of each agent was inspected by the naked eye. The interaction of the combination was classified as follows: synergistic when the fractional inhibitory concentration index (FICI) was ≤ 0.5; additive when FICI was > 0.5and ≤ 1; indifferent when FICI was > 1 and ≤ 4; and antagonistic when FICI was > 4 (Barchiesi et al., 1998).

### 2.3 Antibiofilm assay

To determine whether the combination of dioscin and AmB also produces a synergistic effect on biofilm formation, a checkerboard assay was performed within the framework of the biofilm formation assay. Freshly prepared *C. albicans* SC5314 cell suspension in RPMI 1640 medium (10^6^ cells/mL) was added to 96-well plates, followed by the addition of dioscin (0.125–8 μg/mL) and AmB (0.156–10 μg/mL) in a twofold serial dilution. Wells containing only medium were set as the blank control, while wells containing fungal suspension plus the same volume of dimethyl sulfoxide (DMSO) were set as the negative control. After 24 h of growth in the presence of different drug combinations, the biofilms were washed, and 100 μL XTT solution was added to each well. After a 2-h incubation in the dark, the supernatants (70 μL) were transferred to a new plate. The optical density (OD) of each well at 490 nm (OD_490_) was detected with a multifunctional plate reader (VarioSkan, Thermo, Vantaa, Finland). The viability of fungal biofilms in each well was calculated as follows: viability = (OD_490 treatment_ − OD_490 blank_)/(OD_490 control_ – OD_490 blank_) (Yang et al., 2018b).

After the synergistic effects on biofilm formation were confirmed, specific concentrations of dioscin (1 μg/mL) and AmB (0.625 μg/mL) were selected for further assays. This combination was then tested on both biofilm formation and biofilm development. For tests on biofilm development, biofilms formed in the absence of any drugs were washed with phosphate-buffered saline (PBS) and exposed to fresh RPMI 1640 medium containing no drug, dioscin (1 μg/mL), AmB (0.625 μg/mL), and dioscin (1 μg/mL) plus AmB (0.625 μg/mL). After 24 h, the biofilms were washed and subjected to the XTT assay, as described earlier. These assays were performed in triplicate and repeated three times.

### 2.4 Visualization of the synergistic effects of dioscin and AmB on biofilm formation

To show the direct effects of this combination on biofilm formation, *C. albicans* biofilms formed in the presence of control (DMSO), dioscin (1 μg/mL), AmB (0.625 μg/mL), and dioscin (1 μg/mL) plus AmB (0.625 μg/mL) were stained with calcofluor white (CFW; final concentration: 20 μg/mL) and washed with PBS, followed by confocal laser scanning microscope (CLSM; Olympus FV3000 Tokyo, Japan) analysis. The recordings of biofilms were performed in a 3D scanning mode under a × 40 objective, with a step size of 2 µm. The reconstruction was performed with Imaris (version 7.2.3).

### 2.5 Adhesion assay

*C. albicans* SC5314 cells from an overnight culture were prepared in RPMI 1640 medium to get a density of 10^6^ cells/mL. Such suspensions were transferred into 96-well plates and exposed to control (0.5 μL DMSO), dioscin (1 μg/mL), AmB (0.625 μg/mL), and dioscin (1 μg/mL) plus AmB (0.625 μg/mL) at 37 °C for 1.5 h. Wells containing medium only were used as the blank control. PBS washing and XTT reduction assay, as described earlier, were then performed to detect the relative viability of the adherent cells remaining on the bottoms of the plates. The relative adhesion of each treatment was calculated as follows: (OD_490 treatment_ − OD_490 blank_)/(OD_490 control_ − OD_490 blank_).

### 2.6 Hyphal assay

*C. albicans* cells in RPMI 1640 medium (10^6^ cells/mL), obtained through dilution from overnight YPD cultures in which the *Candida* cells were mainly in the yeast form, were exposed to control (DMSO), dioscin (1 μg/mL), AmB (0.625 μg/mL), and dioscin (1 μg/mL) plus AmB (0.625 μg/mL) at 37 °C for 4 h. *Candida* cells in the absence of hyphal growth-inhibiting agents grew into hyphae, whereas agents inhibiting hyphal growth caused reduced hyphae formation or shorter hyphae. Cell morphologies were observed under an inverted microscope (objective: × 40).

### 2.7 Time-killing assay

In this assay, freshly prepared *C. albicans* SC5314 suspensions in RPMI 1640 medium (at a density of 10^6^ cells/mL) were exposed to DMSO (control), dioscin (1 μg/mL), AmB (0.625 μg/mL), and dioscin (1 μg/mL) plus AmB (0.625 μg/mL). The suspensions were incubated at 28 °C, with rotation at 140 rpm. At 0, 2, 4, 6, 8, 12, and 24 h, 100 μL cultures were fetched from each treatment for 10 × serial dilution and spreading on Sabouraud dextrose (SD) agar plates. The number of colony-forming units (CFU) on each agar plate was counted manually after the plates were incubated at 37 °C for 48 h.

### 2.8 Reactive oxygen species assay

AmB was shown to induce reactive oxygen species (ROS) in *C. albicans* cells (Guirao-Abad et al., 2017), while dioscin can facilitate excessive ROS production in cancer cells (Zheng et al., 2022), and rimonabant can potentiate AmB by elevating cellular oxidative stress in *C. albicans* (Zhang et al., 2021). Based on these findings, we tested whether dioscin can enhance the ROS production caused by AmB. Fresh *C. albicans* suspensions in 96-well plates were treated with control (DMSO), dioscin (1 μg/mL), AmB (0.625 μg/mL), and dioscin (1 μg/mL) plus AmB (0.625 μg/mL) for 4 h at 37 °C. Cells were then stained with DCFH-DA (Solarbio, Beijing, China; final concentration: 10 μM) in the dark for 20 min. After washing with PBS three times to remove excessive dye, fungal cells were sent to CLSM (Olympus FV3000, Japan) for ROS detection, under a × 40 objective. The excitation wavelength of the laser used was 488 nm.

### 2.9 PI staining

The freshly prepared *C. albicans* SC5314 suspensions (in RPMI 1640 medium, 10^6^ cells/mL) were transferred into wells of a 96-well plate and challenged with control (DMSO), dioscin (1 μg/mL), AmB (0.625 μg/mL), and dioscin (1 μg/mL) plus AmB (0.625 μg/mL), for 4 h. The fungal cells were then stained with PI (stock solution: 1 mg/mL in PBS; final working concentration: 10 μg/mL) for 10 min. After washing with PBS, the cells were sent for CLSM analysis under a × 40 objective, using the laser at 561 nm.

### 2.10 DiBAC_4_(3) staining

To detect whether the cell membrane potential was affected by dioscin and AmB, the voltage-sensitive fluorescent dye DiBAC_4_(3) (stock solution: 10 mM in DMSO; final concentration: 1 μM) was used to stain *C. albicans* cells. This assay was performed in 96-well plates. Fungal cells (at a density of 10^6^ cells/mL in RPMI 1640 medium) were exposed to control (DMSO), dioscin (1 μg/mL), AmB (0.625 μg/mL), and dioscin (1 μg/mL) plus AmB (0.625 μg/mL) for 4h at 37 °C. The fungal cells were then stained with DiBAC_4_(3) for 10 min. After washing with PBS to remove the residual dye DiBAC_4_(3), samples were observed with CLSM under a × 40 objective using the laser at 488 nm.

### 2.11 Scanning electron microscope and transmission electron microscope

To exclude the interference of cellular morphologies that may be caused by the RPMI 1640 medium at 37 °C, *C. albicans* cells were cultured in YPD medium in scanning electron microscope (SEM) and transmission electron microscope (TEM) assays. For SEM analysis, *C. albicans* cells (10^6^ cells/mL) were treated with DMSO (control, same volume as in the treatment with dioscin plus AmB) or dioscin (4 μg/mL) plus AmB (2.5 μg/mL) at 37 °C for 4 h, followed by centrifugation and fixation in 2.5% glutaraldehyde at 4 °C overnight. Samples were then transferred to Huiceshi Company (Suzhou, China) for SEM scanning using a Quattro S system (Thermo Scientific, Eindhoven, Netherlands) under a × 10,000 magnification. Samples were scanned in a high-vacuum mode with a voltage of 15 kV; the spot size was 2.0, and the working distance (WD) was 9.1 mm.

For TEM analysis, *C. albicans* cells (10^6^ cells/mL) were treated with DMSO (control) or dioscin (4 μg/mL) plus AmB (2.5 μg/mL) at 37 °C for 16 h, and then the cells were centrifuged at 3,000×*g* for 5 min and fixed in 2.5% glutaraldehyde at 4 °C overnight. Samples were sent to Servicebio Inc. (Wuhan, China) for desiccation, embedding (SPI-Pon™ 812, SPI Supplies, West Chester, USA), and ultrathin section (Leica UC7, Leica Microsystems, Wetzlar, Germany) at 80 nm thickness. Sections were placed on copper grids and stained with 2% uranyl acetate for 8 min and then with lead citrate for 8 min, and photographed subsequently with a Hitachi TEM system (HT7800, Hitachi, Tokyo, Japan) under a ×10,000 magnification. The photos were obtained in a high-contrast mode with an accelerating voltage of 80 kV.

### 2.12 Statistical analysis

The results shown here were mean ± standard deviation from triplicates in three independent assays, and Student’s *t*-test (by GraphPad Prism 6.02) was used to analyze the statistical significance between drug-free controls and treatments. *p* < 0.05 was considered significant.

## 3 Results

### 3.1 Drug interactions in *Candida* spp. planktonic growth

As a commonly used method to evaluate the effects of drug combinations, checkerboard assays were employed in the framework of CLSI-M27-A3-guided MIC tests. As shown in **Table 1**, the combination of dioscin and AmB could produce a synergistic effect in the standard strain *C. albicans* SC5314 and in the fluconazole-resistant strain *C. albicans* ATCC10231. Although this combination did not produce synergistic interactions in *C. glabrata* ATCC2001 and *C. parapsilosis* ATCC22019, their interactions were additive. While dioscin did not inhibit the planktonic growth of *C. krusei* ATCC6258 and *C. tropicalis* ATCC7349 (MIC higher than 2,048 μg/mL, as shown in **Table 1**), the combination of dioscin and AmB produced strong synergistic interactions in these two strains (the FICI in these two strains were both below 0.252). The interactions between dioscin and AmB were also tested in four clinical strains. The effects of this combination were synergistic (FICI = 0.5) in two isolates, while they were additive in another two isolates (FICI = 0.75). In general, the combination of dioscin and AmB can produce synergistic or additive effects in various *Candida* species and isolates.

**Table 1:** Interaction of dioscin with AmB in various *Candida* strains.

Strains	AmB MIC (μg/mL)(alone/combined)	Dioscin MIC (μg/mL)(alone/combined)	FICI	Interaction
*C. albicans* SC5314	1.25/0.156	4/1	0.375	Synergistic
*C. albicans* ATCC10231	2.5/0.625	4/1	0.5	Synergistic
*C. albicans* isolate 1	2.5/0.625	4/2	0.75	Additive
*C. albicans* isolate 2	2.5/0.625	4/1	0.5	Synergistic
*C. albicans* isolate 3	2.5/0.625	4/2	0.75	Additive
*C. albicans* isolate 5	2.5/0.625	4/1	0.5	Synergistic
*C. glabrata* ATCC2001	2.5/1.25	4/1	0.75	Additive
*C. krusei* ATCC6258	2.5/0.3125	> 2,048/4	< 0.252	Synergistic
*C. tropicalis* ATCC7349	5/0.625	> 2,048/4	< 0.252	Synergistic
*C. parapsilosis* ATCC22019	2.5/0.3125	4/2	0.625	Additive

### 3.2 Biofilm formation and development

**Figure 1:**
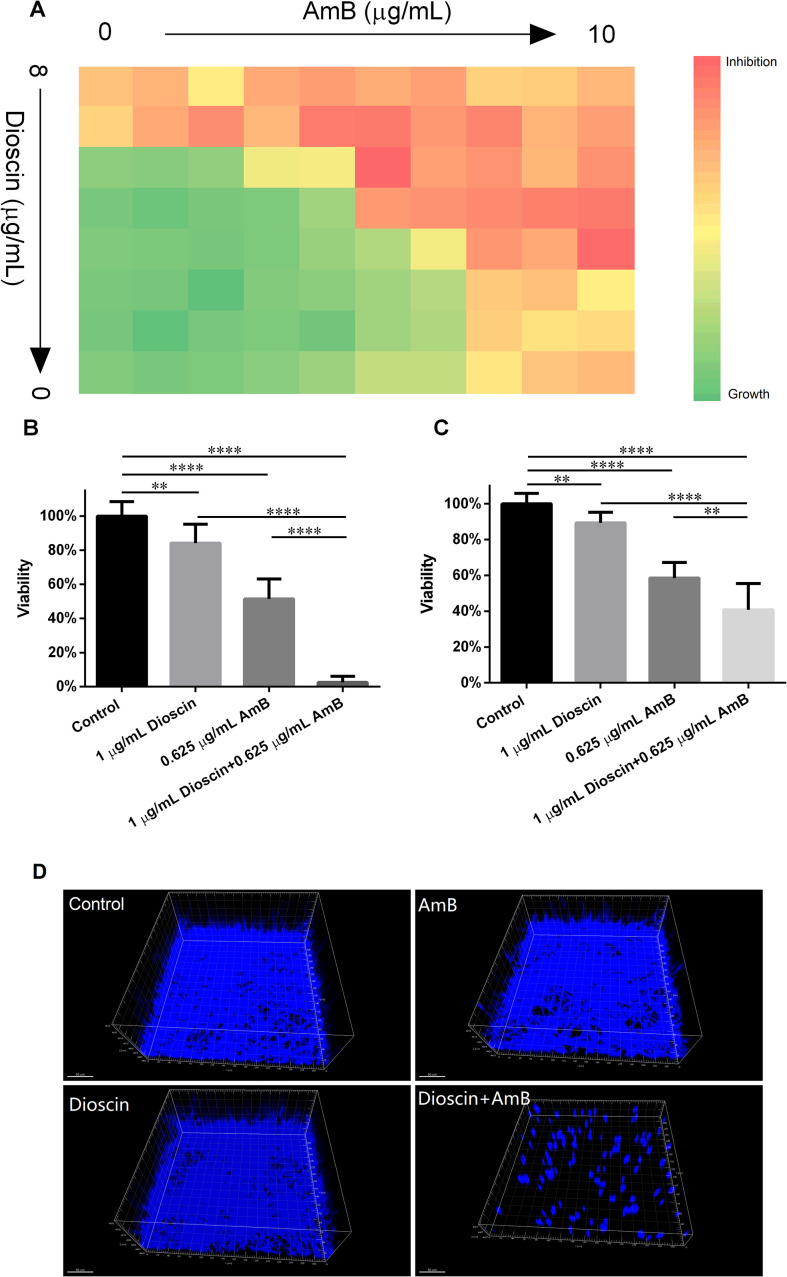
The effects of dioscin and AmB combination on the biofilms of *C*. *albicans* SC5314. **(A)** Heatmap of *C*. *albicans* biofilm growth in the presence of different concentration combinations of dioscin and AmB. The darker green color indicates the higher viability of *C*. *albicans* biofilm, while the darker red/orange color indicates greater inhibition of biofilm growth. Data shown were from an average of three independent assays. **(B)** Dioscin and AmB produced synergistic effects on biofilm viability. **(C)** The development of preformed *C*. *albicans* biofilms could also be suppressed synergistically by dioscin and AmB. ^**^p < 0.01; ^****^p < 0.0001. **(D)** Dioscin (1 μg/mL) and AmB (0.625 μg/mL) produced synergistic effects on the 3D structures of *C*. *albicans* biofilms. Bar: 50 μm.

To test whether dioscin and AmB also have synergistic effects on biofilm formation and development, we also performed a checkerboard assay within the framework of biofilm formation assays. The biofilms exposed to various concentration combinations of dioscin and AmB were subjected to an XTT assay to quantify the inhibition caused by those combinations. As shown in **Figure 1A**, the inhibition of AmB against *C. albicans* biofilm was gradually enhanced by increasing the concentration of dioscin. The combination of dioscin (1 μg/mL) and AmB (0.625 μg/mL) produced a synergistic effect. Thus, this combination was further tested on biofilm formation and development to quantify the inhibitory effects. As shown in **Figure 1B**, the biofilms exposed to dioscin (1 μg/mL) and AmB (0.625 μg/mL) showed only 16% and 49% inhibition on viability, respectively, while biofilms treated with dioscin (1 μg/mL) plus AmB (0.625 μg/mL) inhibited the viability by more than 97%. This confirmed the results from the checkerboard assay (**Figure 1A**). As for preformed biofilms, this combination also showed more suppression than the sum of each group (**Figure 1C**). Dioscin (1 μg/mL) and AmB (0.625 μg/mL) suppressed the development of biofilms by about 11% and 42%, respectively, while dioscin (1 μg/mL) plus AmB (0.625 μg/mL) showed approximately 60% inhibition, further confirming the synergy of this combination in suppressing biofilm development. The results of 3D structures from CLSM recordings also confirmed the synergy between dioscin and AmB in suppressing biofilm formation (**Figure 1D**). The biofilms formed in the presence of dioscin and AmB were much sparser and had fewer and shorter hyphae compared to others.

### 3.3 Time-kill assay

**Figure 2:**
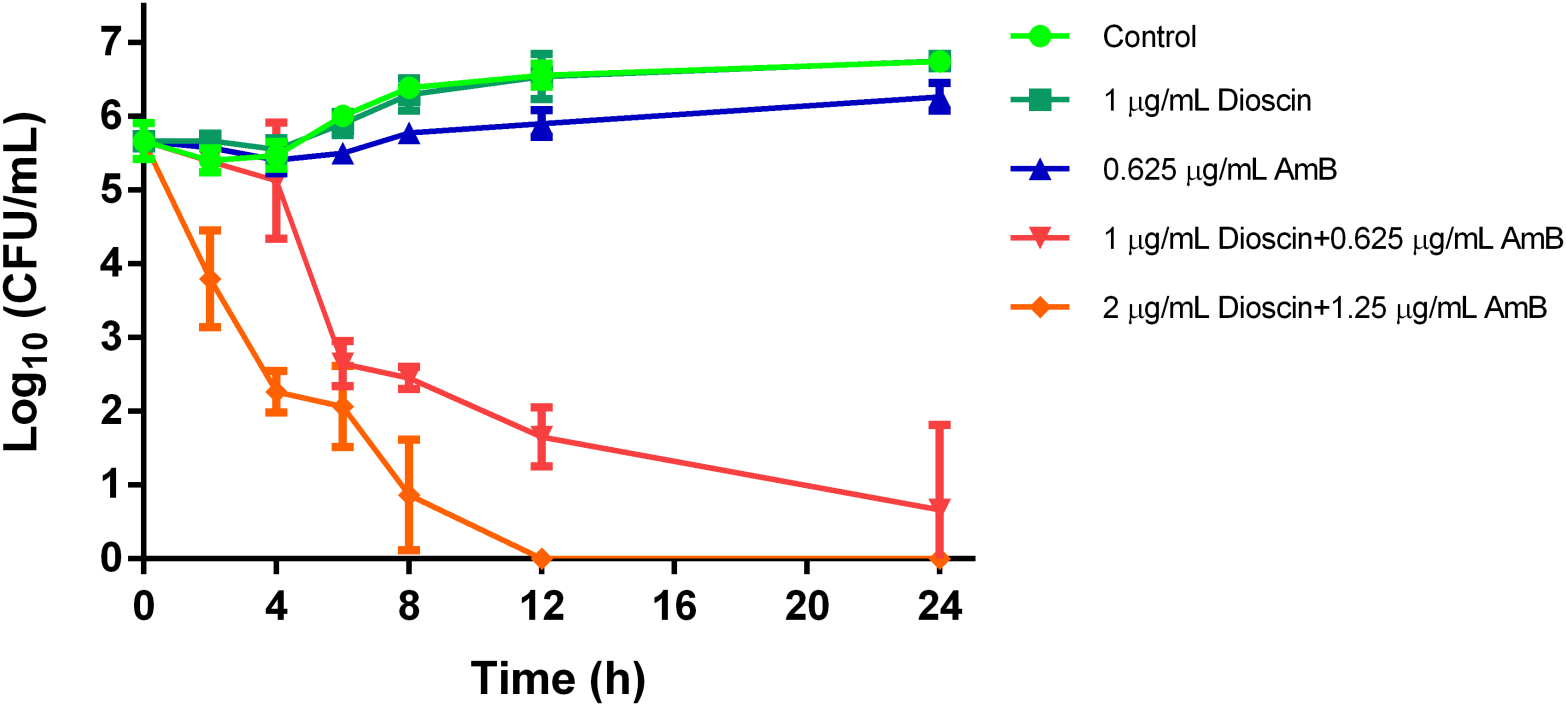
Time-kill kinetics of dioscin, AmB, and the combination. *C. albicans* suspensions in RPMI 1640 medium (10^6^ cells/mL) were cultured with control, 1 μg/mL dioscin, 0.625 μg/mL AmB, 1 μg/mL dioscin + 0.625 μg/mL AmB, and 2 μg/mL dioscin + 1.25 μg/mL AmB for 24 h at 28 °C with a rotation of 140 rpm. At 0, 2, 4, 6, 8, 12, and 24 h, aliquots were taken out, diluted, and spread on SD agar. The CFUs on each agar plate were counted 48 h later.

Time-kill assays were also performed on *C. albicans* SC5314. As shown in **Figure 2**, dioscin + AmB showed potent *Candida-*killing activity after 4 h, as the live fungal cell counts (CFU/mL) dropped 3 log_10_ at 6 h and lowered further as the exposure time increased. The fungicidal effects were more pronounced if the concentrations of dioscin and AmB were both doubled, which obviously lowered the viable *C. albicans* cells from the second hour posttreatment, reaching a 3 log_10_ (CFU/mL) reduction at 4 h. In contrast, the counts of viable cells exposed to 1 μg/mL dioscin were similar to those of the control, while treatment with 0.625 μg/mL AmB only slightly lowered the viable *C. albicans* cell numbers. These killing curves also confirmed the synergistic effects of dioscin and AmB, as well as the long-lasting killing effects of this combination on *C. albicans* (Deng et al., 2025).

### 3.4 Adhesion

**Figure 3:**
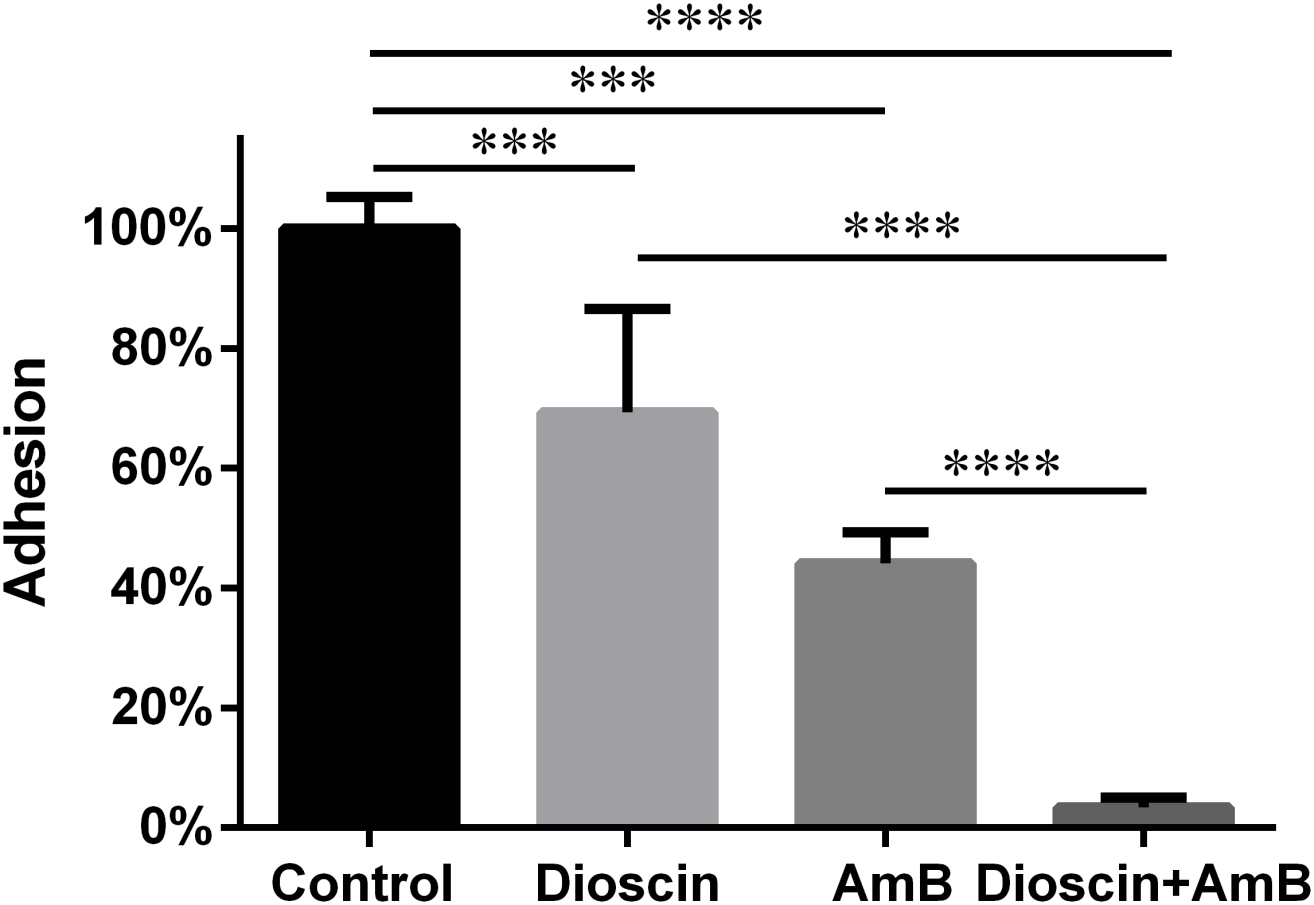
Dioscin and AmB decreased the adhesion of *C. albicans* to polystyrene surfaces. Freshly prepared *C. albicans* cells were challenged with the indicated agents for 1.5 h, followed by PBS washing three times and XTT assay. Dioscin: 1 μg/mL; AmB: 0.625 μg/mL; Dioscin + AmB: 1 μg/mL dioscin + 0.625 μg/mL AmB. ^***^p < 0.001; ^****^p < 0.0001.

As shown in **Figure 3**, although both dioscin and AmB could reduce the adhesion of *C. albicans* cells to polystyrene surfaces of 96-well plate bottoms by about 30% and 55% relative to the drug-free controls, respectively, the combination of dioscin and AmB caused a reduction of about 95% in adhesion. The higher adhesion inhibition of the combination compared with the sum of the inhibition by dioscin and that by AmB further confirmed the synergy between dioscin and AmB.

### 3.5 Hyphal growth

**Figure 4:**
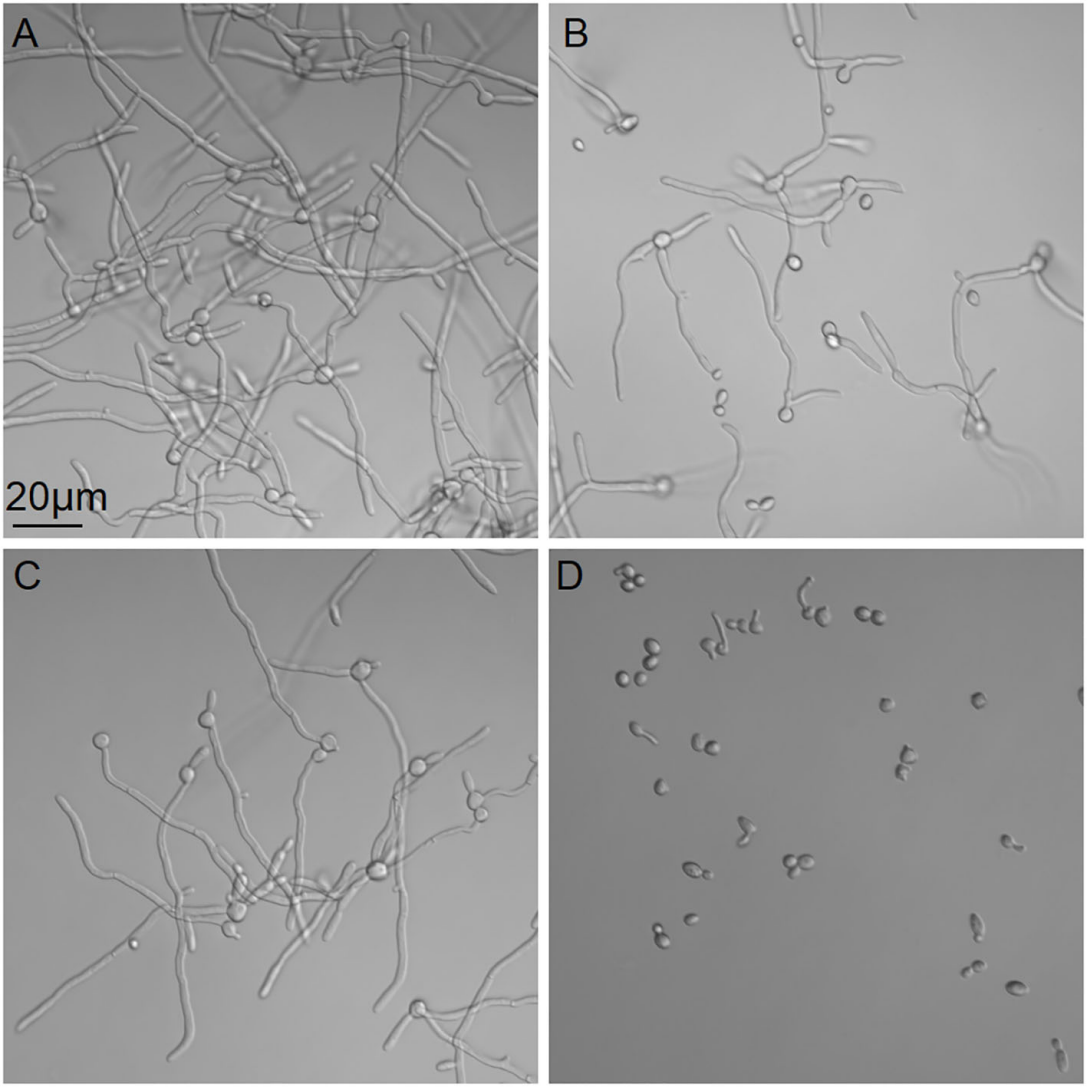
Effects of dioscin and AmB on hyphal growth of *C*. *albicans* SC5314. *Candida* cells were incubated with control **(A)**, 1 μg/mL dioscin **(B)**, 0.625 μg/mL AmB **(C)**, and 1 μg/mL dioscin + 0.625 μg/mL AmB **(D)** at 37 °C for 4 h, followed by microscopic inspection.

The effects of dioscin and AmB on hyphal growth were evaluated in RPMI 1640 medium at 37 °C. As shown in **Figure 4**, 1 μg/mL dioscin alone or 0.625 μg/mL AmB alone could hardly inhibit the growth. However, the combination of both agents could suppress hyphal growth and kept most of the *C. albicans* cells in yeast form. This suggested that dioscin and AmB produced a synergistic effect on inhibiting hyphal growth.

### 3.6 Dioscin does not increase ROS production caused by AmB

**Figure 5:**
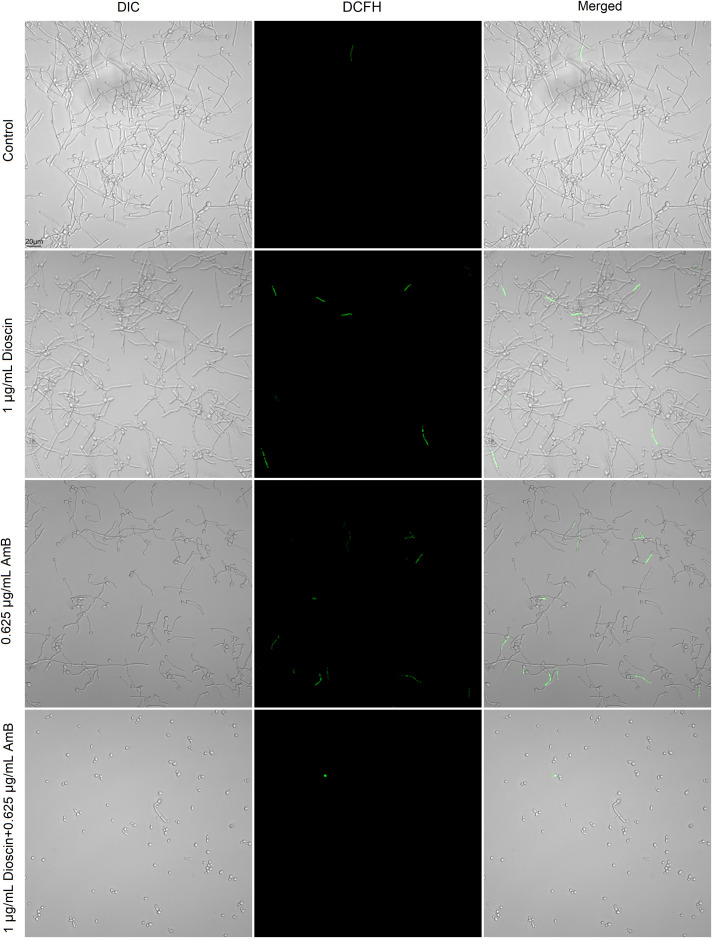
The combination of dioscin and AmB did not cause a significant increase in intracellular ROS production. *C. albicans* cells were treated with the indicated concentration of dioscin, AmB, and both for 4 h at 37 °C, and then they were stained with 10 μM DCFH-DA for 20 min. After washing with PBS, *C. albicans* cells of each group were sent for CLSM to detect the green fluorescence emitted by ROS in fungal cells.

Although AmB can cause ROS production in *C. albicans*, as shown in our results and other reports, we did not notice an obvious rise in intracellular ROS production in cells exposed to either dioscin or AmB. Interestingly, dioscin alone did not induce ROS overproduction in *C. albicans*, although many publications have shown that dioscin can facilitate ROS overproduction in cancer cells. In fact, the combination of dioscin and AmB caused less ROS than AmB (**Figure 5**), indicating that the synergistic effects of this combination were not due to elevated intracellular oxidative stress.

### 3.7 Cell membrane damage

**Figure 6:**
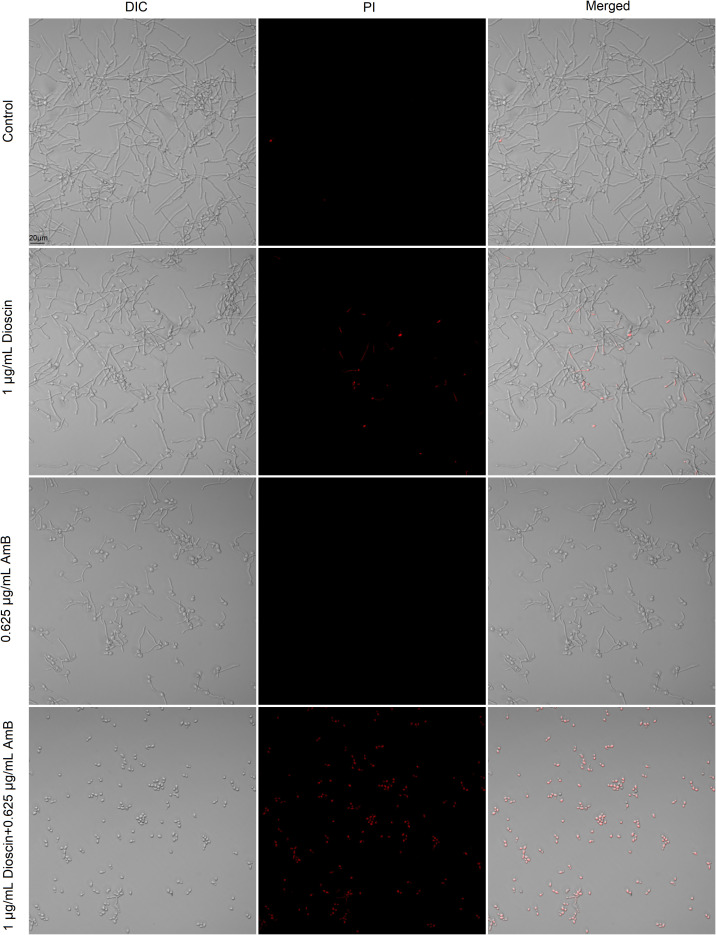
The effects of dioscin and AmB on *C. albicans* plasma membrane permeability. Fungal cells were treated with the indicated concentration of dioscin, AmB, and both for 4 h at 37 °C, and then they were stained with PI for 10 min. After washing with PBS, *C. albicans* cells of each group were sent for CLSM analysis.

To detect the cell membrane damage caused by dioscin and AmB, PI staining was employed. As shown in **Figure 6**, 1 μg/mL dioscin caused more cells to stain with PI compared with the drug-free control, while 0.625 μg/mL AmB almost did not increase cell membrane permeability. In contrast, the combination of dioscin and AmB caused most cells to be stained with PI. This suggested that dioscin and AmB caused much more damage to the cell membrane than either one alone. In other words, dioscin greatly increased the cell membrane damage caused by AmB.

**Figure 7:**
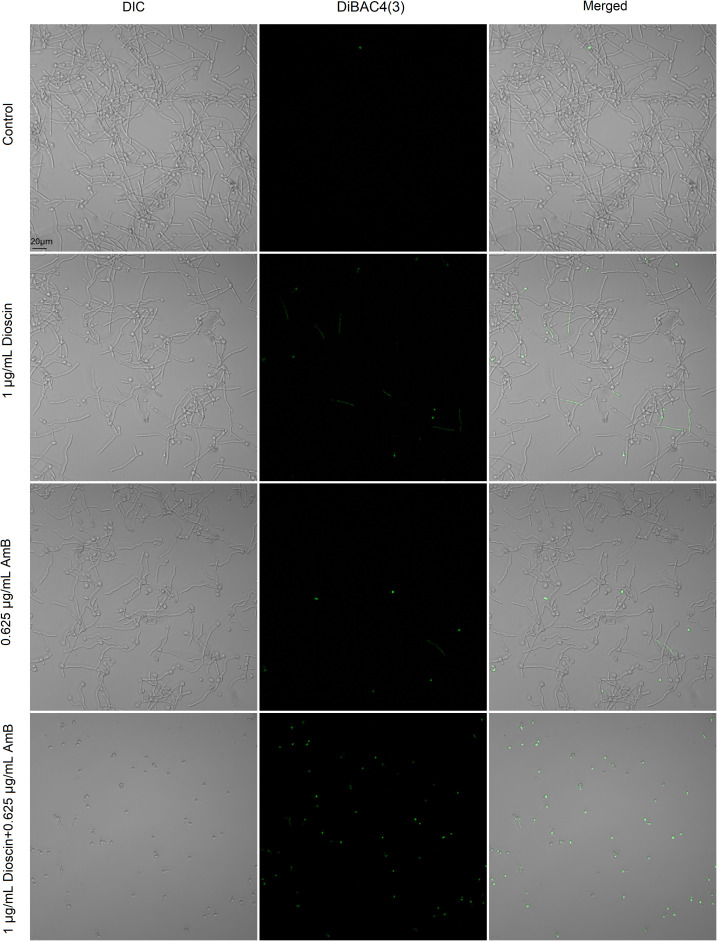
The effects of dioscin and AmB on the plasma membrane potential of *C. albicans*. Fungal cells were treated with the indicated concentration of dioscin, AmB, and both for 4 h at 37 °C, and then they were stained with 1 μM DiBAC_4_(3) for 10 min. After washing with PBS, *C. albicans* cells of each group were sent for CLSM to detect the changes in cell membrane potential.

To further explore the impact of dioscin and AmB on *C. albicans* cell membrane, the voltage-sensitive dye DiBAC_4_(3) was used. As shown in **Figure 7**, cells from the drug-free control were almost not stained by DiBAC_4_(3), indicating no depolarized cells. Some of the cells treated with dioscin or AmB for 4 h showed green fluorescence, indicating depolarized cell membrane potential. The combination of dioscin and AmB caused depolarization of the cell membrane potential in most cells, also showing a synergistic effect.

### 3.8 SEM and TEM analyses

**Figure 8:**
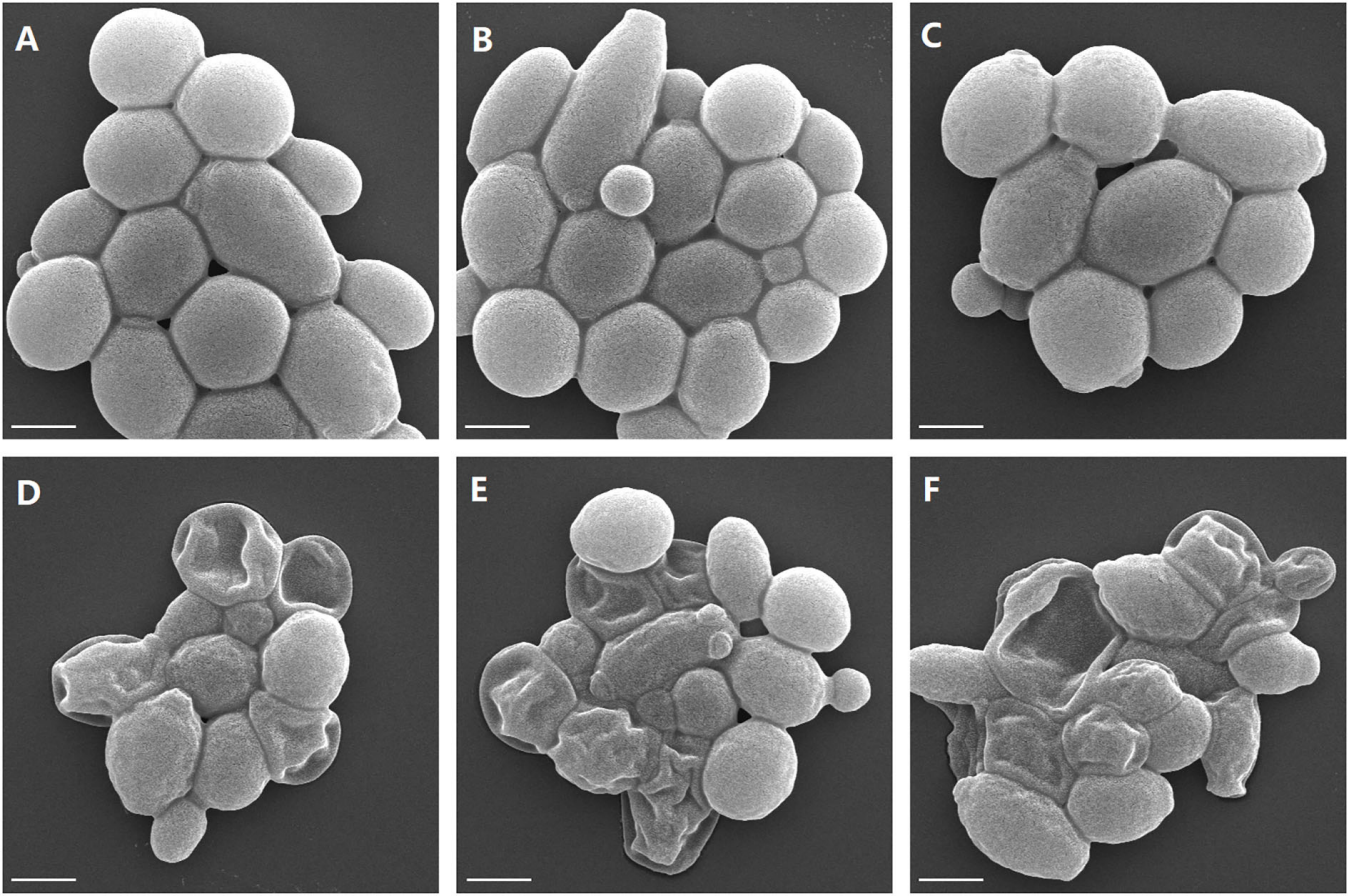
Effects of the combination treatment of dioscin and AmB on *C. albicans* surfaces. **(A–C)** Drug-free control. **(D–F)** Treatment with dioscin (4 μg/mL) and AmB (2.5 μg/mL). Treatment was 4 h, and cells were observed at × 10,000. Bar = 2 μm.

**Figure 9:**
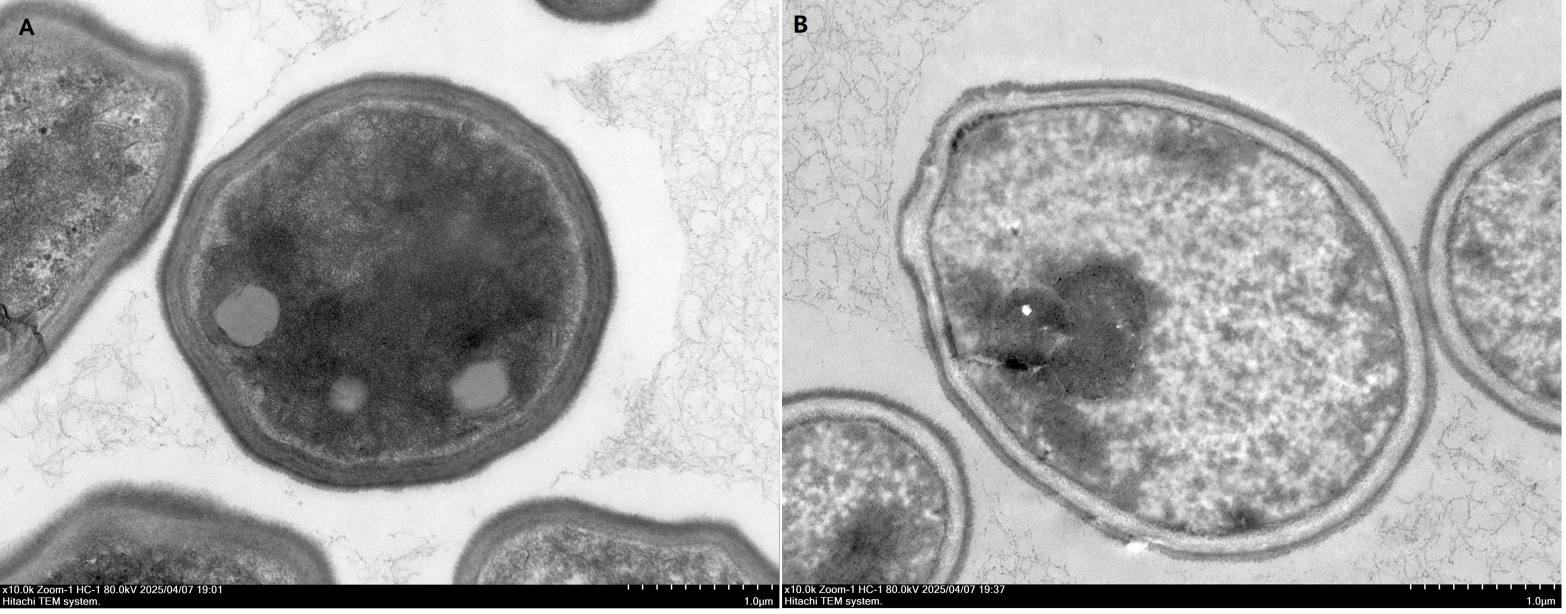
Ultrastructure of *C*. *albicans* cells obtained through TEM. **(A)** Normal *C*. *albicans* cells. **(B)** Treatment with dioscin (4 μg/mL) and AmB (2.5 μg/mL). Cells were observed under TEM at × 10,000 magnification.

We also performed SEM analysis to show the changes in cell ultrastructure. As shown in **Figure 8**, *C. albicans* cells without drug treatment showed round or ovoid shapes and had smooth surfaces (**Figures 8A–C**). Treatment with dioscin and AmB for 16 h caused severe cellular changes, and rough cell surfaces could be seen. Even the collapse of cells was observed in dioscin plus AmB-treated cells (**Figures 8D–F**), which may indicate the loss of intracellular materials. This may suggest damage to the cell membrane, although cell membrane changes could not be observed directly by SEM. Under TEM (**Figure 9**), treatment with dioscin and AmB caused the loss of intracellular materials.

## 4 Discussion

In the case of resistance development, a fitness cost would be paid by yeasts, causing vulnerability to other environmental stimuli. It seems that resistance to agents targeting single proteins develops more frequently than resistance to membrane-acting agents (Revie et al., 2022). Combination therapy can have elevated efficacy and a lower possibility of developing resistance (Ejim et al., 2011). A good example is the combined use of AmB and 5-flucytosine in treating cryptococcal meningitis, which has a mortality of 100% in untreated patients and claims 181,000 deaths per year (Iyer et al., 2021). The combined use of fluconazole and 5-flucytosine was also demonstrated to prevent the appearance of resistant *Cryptococcus* spp. in patients’ cerebrospinal fluid (Stone et al., 2019).

Dioscin is a membrane-active saponin that is derived from many herbal plants, such as *Dioscorea nipponica* and *Dioscorea cayenensis* (Yang et al., 2018a). Although dioscin has been reported by several groups to have antifungal activities against *C. albicans*, as well as other *Candida* species (Barros Cota et al., 2021; Cho et al., 2013; Yang et al., 2018a), the interactions between dioscin and AmB have never been explored. In this study, the influence of dioscin and AmB on *C. albicans* and its virulence factors was explored.

The combination of dioscin and AmB not only demonstrated significant synergistic effects on the planktonic growth of *C. albicans* standard strains and clinical isolates but also showed strong synergies in *C. krusei* ATCC6258 and *C. tropicalis* ATCC7349, where the MICs were higher than 2,048 μg/mL. The time-kill kinetics of the combination also confirmed the synergy. In other words, dioscin potentiated the fungicidal activity of AmB.

Hyphae of *C. albicans* present a major attribute critical for epithelial damage and translocation from intestinal tracts to blood vessels (Allert et al., 2018). In addition, *C. albicans* hyphae are an integral part of biofilm and can fortify their structure (Liu et al., 2017). As a physiological adaptation to a microbial lifestyle on biotic or abiotic surfaces, biofilms formed by *C. albicans* could confer resistance and tolerance to antifungal drugs, enabling survival in the presence of antifungal drugs (Gow et al., 2022). The combination of dioscin and AmB can significantly inhibit hyphal formation and biofilm formation. Biofilm-associated infections are usually difficult to treat. In the development of formed biofilms, the activity of AmB can also be potentiated by dioscin, suggesting that this combination might be employed as a candidate therapy for treating *C. albicans* biofilm-associated infections.

Dioscin can induce ROS production in eukaryotic mammalian cells (Yao et al., 2020; Zhao et al., 2016), and AmB has also been reported as a ROS-inducing antifungal (Guirao-Abad et al., 2017). However, in this study, we did not detect an obvious increase in intracellular ROS production. In contrast, the ROS level in cells exposed to AmB plus dioscin was less than that in cells challenged with dioscin or AmB. This indicates that ROS production might not be the primary mechanism underlying this combination.

Both dioscin and AmB were reported to be membrane-disruptive antifungal agents (Cho et al., 2013; Yang et al., 2018a). Therefore, we proposed that this combination may cause more serious damage to cell membranes. The results from the cell membrane permeability assay (PI staining) and cell membrane potential assay (DiBAC_4_(3) staining) confirmed our speculation. This may be the case described by others, which showed that the combination of two drugs whose targets converge on the same essential biological function can lead to synergy, similar to the synthetic lethal interaction of two genes (Spitzer et al., 2016). The synergy between two antifungal agents is common, such as amphotericin B and echinocandins in treating *Candida auris* (Hernando-Ortiz et al., 2024), artemisinin and AmB in *C. albicans* (Zhu et al., 2021), ribavirin and fluconazole in *C. albicans* (Zhang et al., 2020), as well as 4-(5-methyl-1,3,4-thiadiazole-2-yl) benzene-1,3-diol and AmB in multiple *Candida* species (Chudzik et al., 2019). Our results expanded further these combinations, providing another potential choice in dealing with *C. albicans* infections.

One of the adverse effects of AmB is the toxicity to the liver and kidney, while dioscin has demonstrated protective effects on the liver and kidney in multiple publications (Li et al., 2021; Liu et al., 2021; Xu et al., 2014; Yang et al., 2019b; Zheng et al., 2018). Especially in damage induced by drugs, such as alcohol (Xu et al., 2014), thioacetamide (Zheng et al., 2018), dimethylnitrosamine (Zhang et al., 2016), methotrexate (Li et al., 2021), and doxorubicin (Song et al., 2019), dioscin has shown significant protection for the liver. In animal models of renal injuries caused by fructose (Qiao et al., 2018), doxorubicin (Zhang et al., 2017b), and cisplatin (Zhang et al., 2017a), dioscin also showed significant protection. Given these protective effects of dioscin demonstrated on the liver and kidney, the combination of dioscin and AmB likely has true dual effects on fungal infections: potentiating the therapeutic efficacy of AmB and lowering its toxicity.

## 5 Conclusion

In sum, our study demonstrated the *in vitro* synergy of dioscin and AmB against *C. albicans* standard strains and clinical isolates, as well as other *Candida* species. When dioscin and AmB were used in combination, the fungicidal activity, hyphal inhibition, suppression of biofilm formation, and development could be greatly enhanced compared to either agent alone. However, the combination did not cause excessive ROS production, although AmB has been reported to increase intracellular ROS. The cell membrane damage caused by this combination was more severe than that caused by either agent alone, as revealed by elevated cell membrane potential depolarization and cell membrane permeability. Our results suggest that dioscin and AmB may be a promising combination candidate for developing therapies against *C. albicans* infections.

## Data Availability

The raw data supporting the conclusions of this article will be made available by the authors, without undue reservation.
